# Unsupervised free-breathing 3D imaging of morphology, function and flow in congenital heart disease

**DOI:** 10.1186/1532-429X-16-S1-P134

**Published:** 2014-01-16

**Authors:** Rajesh Krishnamurthy, Ramkumar Krishnamurthy, LaDonna Malone, Elijah Bolin, Amol Pednekar

**Affiliations:** 1 Radiology, TexasChildren's Hospital, Houston, Texas, USA; 2 Pediatrics, Baylor College of Medicine, Houston, Texas, USA; 3 Research Scientist, Philips Medical Systems, Houston, Texas, USA

## Background

To evaluate technical feasibility, image quality and quantitative integrity of a free-breathing protocol following administration of blood pool contrast agent, utilizing 3-dimensional (3D) imaging of morphology, function, and flow without physician supervision in a cohort of patients with CHD.

## Methods

Five patients with CHD were included in this pilot study. The MR studies were performed on a Philips Acheiva 1.5T magnet using a multielement phased array coil with the following sequences in this order: 1. Free-breathing, respiratory synchronized [1], time-resolved MRA following injection of 0.03 mmol/kg of Gadofosveset, and injection rate 2-4cc/second using a power injector. Duration: < 1 minute 2. Free breathing equilibrium phase MRA, acquired voxel size 0.8 × 0.8 × 1.6 mm, 2 NEX. Duration: 2.5-4 minutes 3. Free breathing 3D cine SSFP with respiratory triggering (TR/TE/flip angle: 3/1.5/60; acquired voxel size: 1.5-1.9 × 1.5-2.1 × 7-8 mm3; SENSE acceleration factor: 1.5-2 × 1.5-2; temporal resolution: 30-45 ms). Duration: 4.5-7 minutes 4. Free breathing sagittal 4D phase contrast (PC) imaging with respiratory navigator (18-26 phases/cardiac cycle, Venc 150 cm/sec, spatial resolution 1.6-2.8 mm3.) Duration: 6-12 minutes 5. Free breathing 3D SSFP with respiratory navigator. (acquired voxel size 1 × 1 × 2 mm3) Duration: 5-7 minutes Comparative data was obtained using conventional 2D cine respiratory triggered SSFP sequences (2) in the VLA, 4 chamber and short axis planes, and 2D PC imaging. Data Analysis: Image quality assessment and quantitative volumetric and flow analysis was performed by a single blinded user. MRA images were graded using a semi-quantitative scale from 1-5 for relevant imaging targets in CHD [1], with 1: excellent, no limitations, and 5: non-diagnostic. The clinical scoring system for 2D and 3D cine SSFP was based on blood-myocardial contrast, endocardial edge definition and inter-slice alignment[2]. Paired t-test analysis was performed on LV and RV volumes.

## Results

All free-breathing 3D sequences were technically feasible in all 5 patients. Average time for performance of 5 free breathing 3D sequences was 29 minutes. Average score for first-pass MRA was 1.9/5. Average score for equilibrium MRA was 1.3/5. Clinical scores for 2D SSFP were consistently better than 3D-SSFP, but 3D SSFP images were adequate for recognition of pathology in all cases. Average percentage difference between 2D and 3D cine SSFP volumetric data were as follows: LVEDV (5.5%), LVSV (11.2%), LVEF (6%), RVEDV (-3.9%), RVSV (8%) and RVEF (9.1%). Comparative flow analysis between 2D PC and 4D PC data is pending.

## Conclusions

It is feasible to perform an observer independent comprehensive CMR in CHD utilizing free-breathing 3D acquisitions for morphology, function and flow within 30 minutes.
